# N-Acetyl Cysteine Protects against Methamphetamine-Induced Dopaminergic Neurodegeneration via Modulation of Redox Status and Autophagy in Dopaminergic Cells

**DOI:** 10.1155/2012/424285

**Published:** 2012-09-27

**Authors:** Prashanth Chandramani Shivalingappa, Huajun Jin, Vellareddy Anantharam, Anumantha Kanthasamy, Arthi Kanthasamy

**Affiliations:** Parkinson's Disorder Research Laboratory, Iowa Center for Advanced Neurotoxicology, Department of Biomedical Sciences, College of Veterinary Medicine, Iowa State University, Ames, IA 50011, USA

## Abstract

Methamphetamine- (MA-) induced neurotoxicity is associated with mitochondrial dysfunction and enhanced oxidative stress. Our previous study demonstrated that MA induces autophagy in a dopaminergic neuronal cell model (N27 cells). The cellular mechanisms underlying MA-induced autophagy and apoptosis remain poorly characterized. In the present study we sought to investigate the importance of GSH redox status in MA-induced neurotoxicity using a thiol antioxidant, N-acetylcysteine (NAC). Morphological and biochemical analysis revealed that MA-induced autophagy in N27 dopaminergic cells was associated with pronounced depletion of GSH levels. Moreover, pretreatment with NAC reduced MA-induced GSH depletion and autophagy, while depletion of GSH using L-buthionine sulfoximine (L-BSO) enhanced autophagy. Furthermore, treatment with NAC significantly attenuated MA-induced apoptotic cell death as well as oxidative stress markers, namely, 3-nitrotyrosine (3-NT) and 4-hydroxynonenal (4-HNE). Together, these results suggest that NAC exhibits significant protective effects against MA-induced dopaminergic cell death, presumably via modulation of the GSH level and autophagy. Collectively, our data provide mechanistic insights into the role of cellular GSH redox status in MA-induced autophagy and apoptotic cell death, and additional studies are needed to determine the therapeutic effectiveness of cellular redox modifiers in attenuating dopaminergic neurodegeneration *in vivo*.

## 1. Introduction

Methamphetamine (MA) is a highly addictive psychostimulant that has been shown to cause potent central nervous system stimulant effects. Abuse of this psychostimulant has become an international public health problem, with an estimated 15-16 million users worldwide [1]. MA-induced euphoric effects are accompanied by decreased appetite, hypothermia, paranoia, aggression, and a heightened sense of pleasure [2]. MA neurotoxicity is characterized by long-term reductions in dopaminergic and serotonergic functions, including depletion of dopamine transporter (DAT), serotonin transporter (SERT), serotonin (5-HT), and dopamine (DA) [1]. Magnetic resonance imaging of the brain has shown that chronic use of MA causes neuronal damage [3]. MA abuse has also been linked to increased risk of developing Parkinson's disease (PD). Despite the extensive evidence that substituted amphetamines are neurotoxic, the exact mechanism of action remains poorly understood. A growing body of evidence suggests that MA-induced neurotoxicity involves reactive oxygen species (ROS) and reactive nitrogen species (RNS) [4] and activation of downstream oxidative stress mechanisms. MA enters dopaminergic neurons via dopamine transporter (DAT) and displaces vesicular dopamine. The displaced amines can be oxidized enzymatically and nonenzymatically to form highly reactive dopamine quinones and reactive oxygen species, leading to enhancement of oxidative stress [5]. In recent years mitochondrial dysfunction has been implicated in the mechanism of MA-induced neurodegeneration [6]. Indeed, exposure to MA decreased mitochondrial membrane potential, increased mitochondrial mass, enhanced protein nitrosylation, and decreased levels of Complexes I, III, and IV of the electron transport chain in primary human cells. Also, antioxidants were found to mitigate the neuronal damage, further suggesting a crosstalk between mitochondrial damage and cellular oxidative stress in MA-induced neurotoxicity [7]. Furthermore, oxidative stress has been observed both *in vitro* and *in vivo*, following MA administration [6, 8, 9].

4-Hydroxy-2-nonenal (4-HNE) is a major oxidative product derived from the breakdown of polyunsaturated fatty acids and related esters [10]. In addition, 4-HNE has been shown to have physiological roles in cell proliferation and differentiation [11] and to cause cellular damage by modification of intracellular proteins [12]. Also, treatment of purified proteins with HNE leads to enzyme inactivation and protein cross linking [13]. Intracellular 4-HNE reacts rapidly with cysteine, lysine, and histidine residues of proteins [14, 15] to form protein adducts. The increases in protein nitration are due to increase in ROS/RNS levels, and 3-nitrotyrosine (3-NT) has served as a marker for the production of reactive nitrogen-centered oxidants (ONOO-, NO_2_, etc.). Nitration of active site tyrosine residues has been shown to alter protein structure and function [16, 17]. Under pathological conditions, 3-NT has been suggested to modify both translational and posttranslational processes [18–20]. Therefore, detection of these two biomarkers (4-HNE and 3-NT) following MA treatment would provide strong evidence for the actual presence of oxidative/nitrative damage.

The brain is especially susceptible to oxidative stress due to its capacity to generate large amounts of reactive oxygen species. Glutathione (GSH), a tripeptide comprised of glutamate, cysteine, and glycine, plays essential roles as antioxidant, enzyme cofactor, cysteine storage, major redox buffer, and neuromodulator in the central nervous system [21]. GSH deficiency has been implicated in neurodegenerative diseases including PD. The earliest events causing neurodegeneration include oxidative stress and mitochondrial dysfunction [22]. Oxidative stress during early PD is associated with dramatic reductions in the cellular antioxidant GSH in the SN. GSH depletion precedes both mitochondrial dysfunction and dopamine depletion and is therefore considered the earliest marker of neurodegeneration [23, 24]. In cell culture models, GSH depletion was associated with increased oxidative stress and decreased mitochondrial function [23, 25]. These results suggest that early loss of GSH in the SN of PD patients could be linked to mitochondrial dysfunction and eventually lead to neurodegeneration. Thus, identification of agents that restore intracellular GSH, which might prevent dopaminergic degeneration in PD, is an important endeavor.

N-acetylcysteine (NAC) is an antioxidant and free radical scavenger that increases intracellular GSH at the cellular level. NAC can act as a precursor for GSH biosynthesis as well as stimulator of the cytosolic enzymes involved in glutathione regeneration [26]. NAC has been shown to protect against 4-HNE-induced neuronal death in cultured granule neurons [27]. Based on NAC's beneficial effects, we hypothesize that NAC may also elicit a protective effect against MA-induced neurotoxicity by modulating oxidative damage. Using an *in vitro* model of MA-induced apoptosis, we investigated the mechanisms of neuroprotection exerted by NAC. Our results revealed that NAC replenishes MA-induced GSH depletion and oxidative and nitrative damage. Most importantly, a partial reduction in LC3-II (marker of autophagy) levels was evidenced in MA/NAC-treated cells, thus highlighting the critical role of oxidative stress mechanisms in MA-induced neurotoxicity and autophagy. Thus, our results demonstrate that alteration of cellular redox status serves as a key trigger not only for the induction of apoptosis but also for autophagy.

## 2. Materials and Methods

### 2.1. Reagents

(+)-Methamphetamine (MA) was kindly provided by NIDA (National Institute of Drug Abuse, Bethesda, MD). Monochlorobimane (via Fluka Analytical), glutathione S-transferase, 3,5-di-tert-butyl-4-hydroxytoulene (via SUPELCO Analytical), dansylcadaverine, buthionine sulfoximine, and antibodies against *β*-actin were purchased from Sigma Chemical Company (St. Louis, MO). N-Acetyl-L-cysteine was purchased from Calbiochem (via EMD Biosciences, Gibbstown, NJ). Antibodies against LC3, 3-nitrotyrosine and 4-hydroxynonenal were obtained from Abcam, Inc., Cambridge, MA. Cell Death Detection ELISA PLUS Assay Kit was purchased from Roche Molecular Biochemicals (Indianapolis, IN).

### 2.2. Cell Culture

The immortalized rat mesencephalic dopaminergic cells (N27 cells) were grown in RPMI 1640 medium supplemented with 10% fetal bovine serum (FBS), 2 mM L-glutamine, 50 units penicillin, and 50 *μ*g/mL streptomycin, referred to as complete RPMI medium hereafter. Cells were grown in a humid atmosphere of 5% CO_2_ at 37°C until they were 70–80% confluent. 

### 2.3. Treatment Paradigm

Confluent cells were harvested and seeded in the density of 0.2–4 × 10^6^/mL. Cells were pretreated with N-acetyl-L-cysteine (NAC) for 1 hour or buthionine sulfoximine (BSO) for 24 hours prior to the treatment with MA for various time points. Treatments were made in a complete RPMI medium.

### 2.4. Determination of Cellular GSH

The monochlorobimane fluorometric method was used to determine the cellular GSH levels. Briefly, treated cells were collected, washed with PBS, and sonicated in a lysis buffer (50 mM Tris, pH 7.4, 5 mM EDTA and 0.001% 3,5-di-tert-butyl-4-hydroxytoulene (BHT)). Samples were centrifuged at 14,000 g for 10 minutes at 4°C. 1 mM of monochlorobimane and 10 U/mL of glutathione S-transferase in 50 mM Tris, pH 7.4, were dissolved and added to the resulting supernatant. 200 *μ*L of the mixture was transferred to a black 96-well plate and incubated at 24°C for 30 minutes. The fluorescence of samples was measured using a SPECTRAmax microplate reader (Molecular Devices Corp., Sunnyvale, CA) with excitation at 485 nm and emission at 645 nm.

### 2.5. Dansylcadaverine Assay

The monodansylcadaverine (MDC) assay to label autophagosomes has been described previously [28]. After treatments, cells were incubated with 0.05 mM MDC in a serum free RPMI medium at 37°C for 30 minutes. Later, cells were harvested and lysed in 10 mM Tris-HCl, pH 7.4, containing 1% Triton X-100. Accumulation of MDC in autophagy vacuoles was measured using a SPECTRAmax microplate reader (Molecular Devices Corp., Sunnyvale, CA) with excitation at 365 nm and emission at 525 nm. The number of cells present in each well was normalized by addition of 0.2 *μ*M ethidium bromide, and the DNA fluorescence was measured with excitation at 530 nm and emission 590 nm. Incorporation of MDC was expressed as specific activity.

### 2.6. Transmission Electron Microscopy

Cells were grown on coverslips and fixed with 2% glutaraldehyde (w/v) and 2% paraformaldehyde (w/v) in 0.1 M sodium cacodylate buffer, pH 7.2, for 48 hours at 4°C. Samples were washed in PBS and then fixed in 1% osmium tetroxide in 0.1 M cacodylate buffer for 1 hour at room temperature. The samples were then dehydrated in a series of graded ethanol, cleared with ultrapure acetone, and embedded using a modified EPON epoxy resin (Embed 812; Electron Microscopy Sciences, Ft. Washington, PA). Resin blocks were polymerized for 48 hours at 70°C. Thick and ultrathin sections were generated using a Leica UC6 ultramicrotome (Leeds Precision Instruments, Minneapolis, MN). Ultrathin sections were collected onto copper grids and images were captured using a JEM 2100 200 kV scanning and transmission electron microscope (Japan Electron Optic Laboratories, Peabody, MA).

### 2.7. Western Blotting

Treated cells were harvested, washed with 1X PBS (pH 7.4), and lysed in RIPA buffer (Sigma) on ice. Samples were sonicated for 15 seconds on ice and centrifuged at 14,000 × g for 20 minutes at 4°C. Supernatants were collected from each sample and separated on 10–15% SDS-PAGE. Proteins were transferred onto nitrocellulose membranes by electroblotting for 90 mins at 4°C under 100 V. Membranes were blocked for an hour and incubated with rabbit polyclonal to LC3B (1 : 4000), mouse monoclonal 3-nitrotyrosine (3-NT) (1 : 1000), and goat polyclonal to 4-hydroxynonenal (4-HNE) (1 : 500) as primary antibodies for overnight at 4°C. For equal protein detection, mouse monoclonal *β*-actin (1 : 5000) was used. Later, the membranes were washed several times and incubated with IR Dye 800-conjugated antirabbit IgG (1 : 5000) or Alexa Fluor 680-conjugated anti-mouse IgG (1 : 10000; Molecular Probes, Invitrogen) as secondary antibodies for an hour at room temperature. Membranes were scanned using the Odyssey IR Imaging system (LICOR) and images were analyzed with Odyssey 2.0 software. 

### 2.8. DNA Fragmentation Assay

Measurement of DNA fragmentation was performed using the Cell Death Detection ELISA PLUS Assay Kit [29]. The procedure was similar to the procedure described in our recent publication [30]. Briefly, cells were resuspended in the lysis buffer and incubated for 30 minutes at room temperature. Lysates were centrifuged at 200 × g for 10 minutes. 20 *μ*L of the supernatant was carefully transferred into the streptavidin-coated microplate and incubated for 2 hours in a mixture of HRP-conjugated antibody cocktail that recognizes the nucleosomes in the sample. After thorough washing of the unbound components, an HRP substrate, ABTS, was added into wells. The final reaction product was measured using a spectrophotometer at 405 nm along with 490 nm as the reference reading.

### 2.9. Data Analysis

Results are presented (PRISM software, GraphPad, San Diego, CA) as fold induction, as compared with the untreated group. Results represent mean ± S.E.M. Statistical analysis was performed by using one-way ANOVA followed by Student Newman–Keuls post-hoc test (PRISM software) in order to compare between groups. *P* values < 0.05 were considered significant.

## 3. Results

### 3.1. MA Induces Autophagy

**Figure 1:**
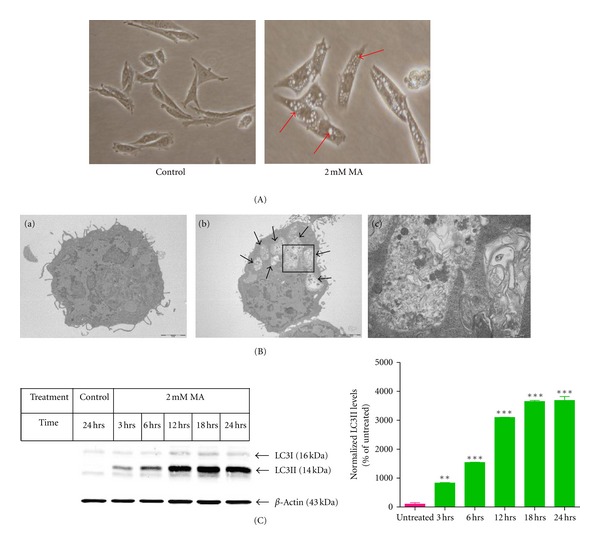
MA induces autophagy. (A) Representative phase contrast microscopy pictures showing abundant cytoplasmic vacuoles (arrows) in N27 dopaminergic cells treated with MA (2 mM) for 12 hours. (B) Representative transmission electron microscopy image analysis of N27 dopaminergic cells exposed to MA (2 mM) for 12 hours: (a) untreated N27 dopaminergic cells; (b) boxed area; (c) autophagosomes observed in N27 dopaminergic cells treated with MA (2 mM) (arrows). Morphology of autophagosomes is characterized by the formation of double membrane vacuoles harboring damaged organelles (arrows) and insoluble protein aggregates. (C) Time-dependent increase in LC3-II levels. N27 dopaminergic cells were exposed to MA (2 mM) for 3, 6, 12, 18, and 24 hours. Equal loading of protein in each lane is confirmed by probing the membrane with *β*-actin antibody. Densitometry analysis of LC3-II induction is represented next to the Western blot image. LC3-II bands were quantified and expressed as percentage of untreated control. Data represent mean ± SEM, *n* = 2. **P < 0.01 and ***P < 0.001 compared with untreated group.

First, we characterized the effect of MA on morphological changes in our mesencephalic dopaminergic neuronal models. As shown in Figure 1(A), 2 mM MA dramatically increased the formation of cytoplasmic vacuoles in N27 dopaminergic cells. The hallmarks of autophagy include the presence of autophagosomes, characterized by double membrane bound vacuoles that contain cytoplasmic material and/or organelles. To examine whether the cell vacuolation induced by MA is related to induction of autophagy, N27 dopaminergic cells were processed after exposure to MA (2 mM) for 12 hours and then ultrastructural analysis was performed using electron microscopy. As shown in Figure 1(B), numerous autophagosomes containing cytoplasmic material and/or organelles were observed in the N27 cells treated with MA (Figure 1(B), b-c).

LC3, an autophagy marker protein, is the mammalian homolog of the yeast ATG8 protein. Upon induction of autophagy, ATG8 protein is covalently modified and redistributed to autophagic vacuoles. In particular, the covalent modification is detected by SDS-PAGE analysis, whereby a shift from LC3-I to LC3-II is evidenced in cells undergoing autophagy [31]. An increase in LC3-II levels was observed starting at 3 hours and reaching a peak at 12 hours following MA treatment (Figure 1(C)). In agreement with electron microscopy analysis, MA treatment increased the expression of LC3-II in a time-dependent manner, thus confirming the formation of autophagosomes during MA neurotoxic insult in dopaminergic neuronal cells.

### 3.2. MA-Induced Suppression of GSH Levels in N27 Cells Is Attenuated by N-Acetylcysteine (NAC)

**Figure 2:**
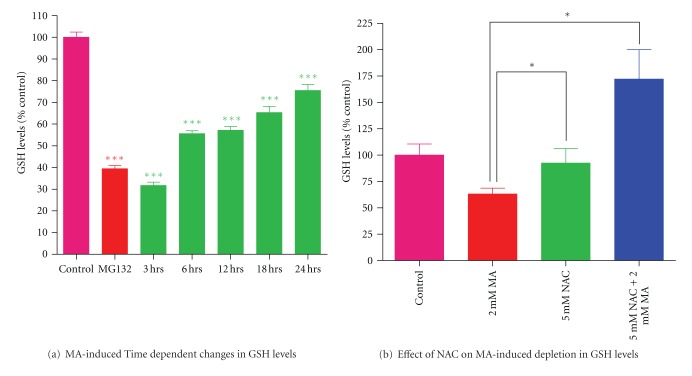
Effect of NAC on MA-induced reduction in total GSH levels. (a) Determination of cellular GSH. N27 dopaminergic cells were treated with 2 mM MA for 3, 6, 12, 18, and 24 hours. MA-induced reduction in GSH levels was measured by the monochlorobimane fluorometric method. The data represent mean ± SEM of six individual measurements. Asterisks (***P < 0.001) indicate significant differences between MA-treated cells and untreated control cells. Values are expressed as percentage of GSH compared with untreated control. Treatment of N27 dopaminergic cells with 5 *μ*M MG132 is considered as positive control. (b) Determination of cellular GSH levels in N27 dopaminergic cells preincubated (1 hour) with 5 mM NAC prior to MA treatment for 18 hours. The data represent mean ± SEM of four individual measurements. Asterisks (*P < 0.05) indicate significant differences between MA-treated cells and NAC alone or NAC with MA-treated cells. Values are expressed as percentage of GSH compared with untreated cells.

Reduced glutathione protects neurons from oxidative damage induced by superoxide, hydrogen peroxide, and other reactive species. Therefore, we examined whether MA alters the GSH levels in dopaminergic cells. As shown Figure 2(a), intracellular stores of GSH were significantly depleted within 3 to 24 hours of MA treatment. While the treatment with 2 mM MA for 3 hours produced a 70% reduction in GSH levels, the 6, 12, 18, 24-hour treatments induced approximately 42%, 40%, 35% and 25% reductions of GHS levels, respectively (Figure 2(a)). Next, we examined whether the GSH precursor NAC can protect cells from MA-induced GSH depletion. N27 dopaminergic cells were pretreated for 1 hour with N-acetylcysteine (5 mM) and then treated for an additional 18 hour with MA (2 mM). MA-induced depletion of GSH levels in N27 cells was attenuated in the presence of NAC, indicating that MA exposure severely compromises the GSH antioxidant redox system.

### 3.3. Effect of NAC and BSO on MA-Induced Autophagy

**Figure 3:**
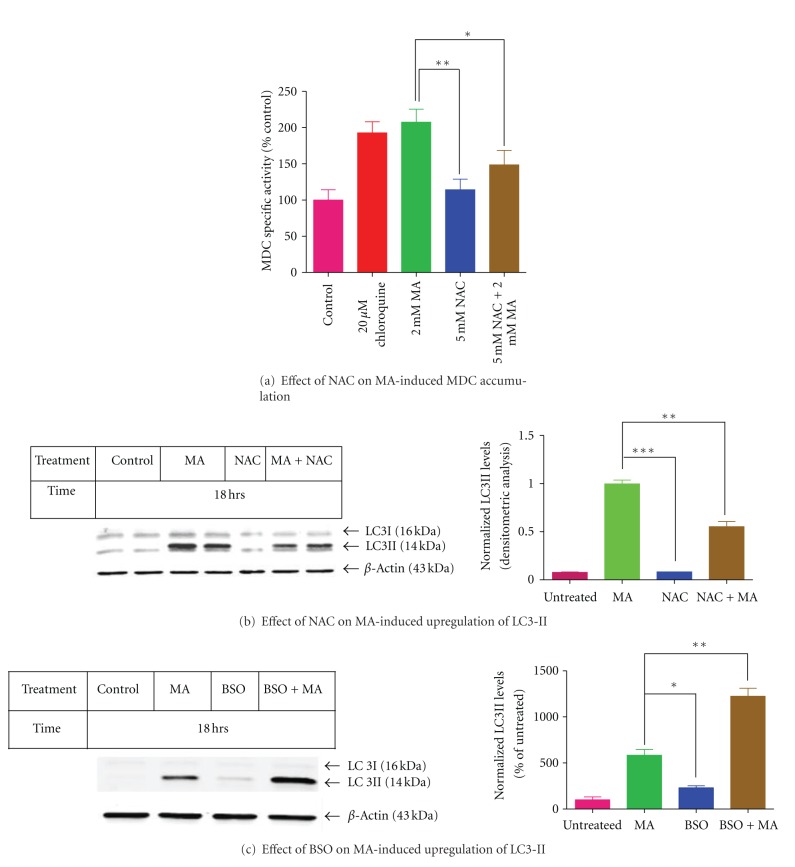
(a) Effect of NAC on MA-induced accumulation of monodansylcadaverine (MDC) in autophagy vacuoles. N27 dopaminergic cells were preincubated with 5 mM NAC for an hour prior to exposure to MA (2 mM) for 18 hours. MA-induced changes in intracellular MDC fluorescence were measured as indicated in Methods. The data represent mean ± SEM of four individual measurements. Asterisks (*P < 0.05 and **P < 0.01) indicate significant differences between MA-treated cells and NAC alone or NAC with MA-treated cells. Values are expressed as percentage of MDC specific activity compared to untreated control cells. Treatment of N27 dopaminergic cells with 20 *μ*M chloroquine is considered as test control. (b) NAC reduced LC3-II levels in N27 dopaminergic cells treated with MA. Western blot analysis of LC3-II expression in N27 dopaminergic cells after exposure to MA (2 mM) with or without 5 mM NAC during 18 hour treatment is presented. Equal loading of protein in each lane is confirmed by probing the membrane with *β*-actin antibody. Densitometry analysis of LC3-II induction (*n* = 2) is represented next to the Western blot image. (c) BSO enhances the expression of LC3-II. N27 dopaminergic cells were pretreated with 100 *μ*M BSO for 24 hours and treated with MA for another 18 hours. Equal loading of protein in each lane is confirmed by probing the membrane with *β*-actin antibody. Densitometry analysis of LC3-II induction is represented next to the Western blot image. LC3-II bands were quantified and expressed as percentage of untreated control. Data represent ± SEM, *n* = 2. *P < 0.05, **P < 0.01, and ***P < 0.001 compared with MA-treated cells.

To investigate the relationship between enhanced GSH and autophagy, we treated N27 cells with NAC, a GSH precursor, and determined the extent of autophagic vacuole formation by MDC fluorescence assay. Pretreatment with NAC dramatically reduced MA-induced MDC accumulation in autophagic vacuoles by approximately 50% (Figure 3(a)). The major limitation of MDC assay is that it labels acidic compartments comprised of endosomal and lysosomal compartments that have recently fused with autophagic vacuoles, namely, late stage autophagosomes and, therefore, results obtained using MDC as a marker for autophagy should be subject to careful interpretation. For this reason, we performed LC3 Western blot analysis to further clarify the role of NAC in MA-induced autophagy.  Figure 3(b) shows NAC (5 mM) treatment partially reversed MA-induced LC3-II expression, further confirming the inhibitory effects of NAC on MA-induced autophagy. In parallel experiments, N27 cells were pretreated for 24 hours with 2 mM L-buthionine-*S*,*R*-sulfoximine (BSO), an inhibitor of GSH biosynthesis, and then were treated for an additional 18 hours with MA (2 mM). MA-induced autophagy was enhanced in the presence of BSO, suggesting that the observed autophagy may be related to the depletion of the endogenous GSH pool. 

### 3.4. Effect of NAC on MA-Induced Increase in Oxidative Stress Markers in N27 Cells

**Figure 4:**
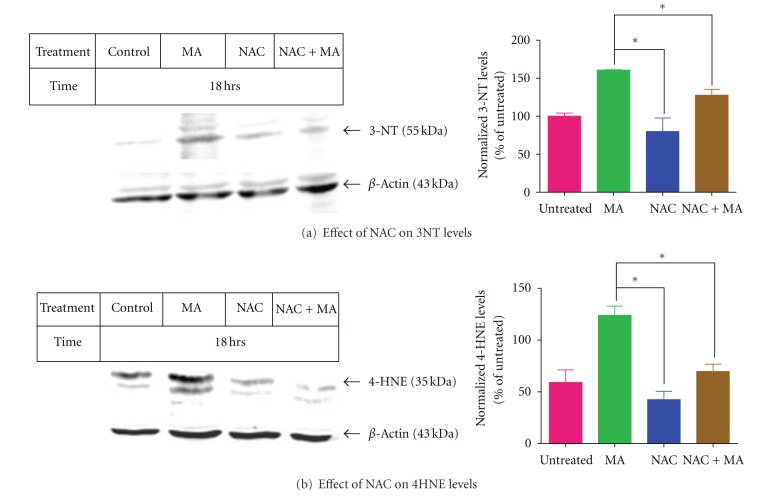
NAC attenuates markers of lipid and protein oxidative damage. (a) Western blot analysis of 3-NT detection and (b) Western blot analysis of 4-HNE detection in N27 dopaminergic neurons preincubated with 5 mM NAC and treated with or without MA (2 mM) for 18 hours. Equal loading of protein in each lane is confirmed by probing the membrane with *β*-actin antibody. Densitometry analysis of 3-NT and 4-HNE induction is represented next to the Western blot image. 3-NT and 4-HNE bands were quantified and expressed as percentage of untreated control. Data represent ± SEM, *n* = 2. *P < 0.05 compared with MA-treated cells.

Peroxynitrite nitrates protein-bound tyrosine residues to produce 3-nitrotyrosine (3-NT). Protein-bound 3-NT was determined by Western analysis using an anti-3-NT antibody.  Figure 4(a) shows that MA increases the level of protein-bound 3-NT compared with the control group, and NAC has an inhibitory effect on MA-induced upregulation of 3-NT. NAC alone had no effect on protein bound 3-NT levels in N27 cells. MA exposure induced upregulation of 4-hydroxynonenal- (4-HNE-) protein adducts, as revealed by Western blot analysis (Figure 4(b)). Such upregulation was reduced by pretreatment with NAC. NAC alone had no effect on 4-HNE levels in N27 cells.

### 3.5. Effects of NAC on MA-Induced Cell Death

**Figure 5:**
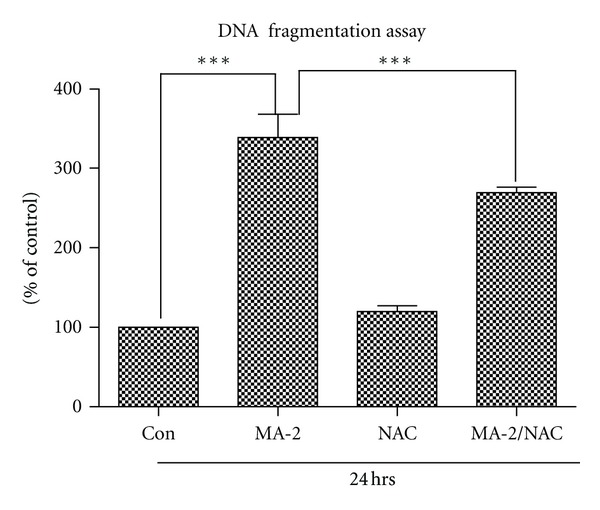
Effect of NAC on MA-induced apoptotic cell death in N27 cells. Cells were pretreated with 5 mM NAC for 1 hour followed by treatment with MA (2 mM) or PBS for 24 hours. DNA fragmentation was quantified using a cell death detection using Roche Elisa PLUS kit. The data are expressed as percentage of DNA fragmentation compared with untreated control cells, and asterisks (***P < 0.01) indicate significant differences between untreated control group with MA-treated group and MA-treated group with NAC group with MA treatment.

We determined MA-induced neuronal apoptosis by DNA fragmentation enzyme-immunoassay. N27 dopaminergic cells treated with 2 mM MA for 24 hours increased by 2-fold DNA fragmentation, as compared to that of the control (Figure 5). To determine whether NAC suppresses MA-induced apoptosis, we treated the cells with NAC for 1 hour prior to MA treatment. While NAC by itself had little or no effect, NAC treatment showed a partial reversal of MA-induced apoptosis (*P* < 0.001). Collectively, these data indicate that NAC can attenuate MA-induced apoptotic death possibly by restoring GSH levels in dopaminergic cells. 

## 4. Discussion and Conclusions

In this study we evaluated the neuroprotective potential of NAC on MA-induced autophagy and apoptosis in the mesencephalic dopaminergic neuronal cell model. We also examined the relationship between cellular redox status, autophagy, and apoptotic cell death following MA exposure. MA produced a substantial reduction in surviving dopaminergic neurons, marked by early depletion of GSH, induction of autophagy, and upregulation of oxidative stress markers, namely, 3-NT and 4-HNE. Indeed, MA-induced oxidative stress has been shown to be a critical event in neurotoxicity. NAC was chosen in this study because of its potent thiol-based antioxidant effect. NAC was able to partially attenuate MA-induced apoptotic cell death, upregulate GSH levels, partially attenuate the autophagy marker LC3-II, and completely abrogate oxidative stress markers. There are several possible mechanisms by which NAC might prevent dopaminergic neuronal cell death. For example, NAC might prevent neuronal cell death via its antioxidant effects capable of reducing reactive oxygen species (ROS). Alternatively, NAC could also enhance intracellular levels of GSH and serve as a reducing agent. Nevertheless, our study highlights the central role of cellular redox status both in the mechanism of neuroprotection and modulation of autophagy. Previous studies have shown that NAC suppresses MA-induced neurotoxicity in striatal neurons [32] and in immortalized human brain endothelial cells [33], but the mechanisms associated with the protective effect were not explored. Our results suggest that NAC treatment restores MA-induced imbalance in cellular redox status and thereby prevents the neuronal cell death. 

The cellular mechanism underlying the proapoptotic effects of MA in dopaminergic neurons remains poorly understood. Multiple mechanisms, including mitochondrial dysfunction, oxidative stress, and apoptosis, have been implicated in MA-induced neurotoxicity [1]. The involvement of oxidative stress in MA-induced neurotoxicity has been studied extensively, whereby accumulation of oxidatively damaged lipids, proteins, and DNA has been shown to occur in the brain regions of animal models as well as in *in vitro* cell culture models of neurodegeneration [34–36]. In fact, oxidative stress has been identified as an early event in dopaminergic degeneration because neurotoxicity has been shown to be attenuated by antioxidants such as Trolox and GSH [37]. Additionally, GSH depletion has also been shown to result in the loss of protein sulfhydryls, including transporter proteins [38]. In line with these findings, we observed that MA-induced apoptotic cell death was preceded by early and pronounced depletion of GSH. Pretreatment of MA-treated cells with NAC restored the GSH levels and decreased apoptotic cell death, indicating that NAC had replenished the GSH levels in these cells, thereby attenuating MA-induced oxidative neuronal cell death. At the cellular level, assessment of reduced GSH is considered to be a marker of cellular antioxidant defense, and a reduction in the levels of GSH is an indicator of oxidative stress [39]. A reduction in GSH alone can act as an inducer of apoptotic events. For example, in a previous report BSO-induced intracellular GSH depletion was found to induce ROS generation and PKC*δ* activation, thereby resulting in cell death in neuroblastoma cells [40]. Also, GSH depletion in a B cell lymphoma cell has been shown to induce ROS-mediated apoptosis [41]. Thus, our study raises the possibility that oxidative stress-dependent GSH depletion may play a role in MA-induced neurotoxicity. The exact nature of MA's effect on endogenous GSH levels remains controversial. For example, MA was found to increase hippocampal, frontocortical, and striatal levels of GSH in both rats and mice [42] following a short treatment with MA; however, other studies found a reduction in striatal GSH after MA administration [43–45]. In a similar fashion, in postmortem brains of MA abusers, a dramatic loss of DA in the caudate was accompanied by a decrease in GSH and increase in GSSG, the oxidized form of GSH [46]. Increasing evidence shows that administration of MA causes a prominent oxidative stress response, which, in turn, leads to severe nigrostriatal dopaminergic neurotoxicity, as evidenced by loss of striatal dopamine transporter (DAT) [1]. In this context, ROS-dependent oxidative stress mechanisms have been suggested in animals that were administered MA [47–51]. Several *in vivo* studies showed the involvement of neuronal nitric oxide synthase (nNOS) in MA-induced neurotoxicity. For example, administration of MA to mice deficient in nNOS or treatment with nNOS pharmacological inhibitors was found to significantly attenuate MA-induced striatal DA and DAT depletion [47, 49]. Other studies demonstrated the overexpression of NOS in the MA-treated mouse striatum [51]. Since both ROS and RNS have very short half-lives, a reliable approach to demonstrate the interaction between nitric oxide and superoxide is the formation of peroxynitrite, which can be determined by measuring the levels of 3-NT residues. In our studies, increased levels of 3-NT following MA suggest that cellular dysfunction is related to excessive production of peroxynitrite. Also, increased production of 3-NT levels during MA treatment positively correlated with cell death. A role for peroxynitrite in MA-induced neurotoxicity has been documented using selenium (a scavenger of two-electron oxidants), which demonstrates a neuroprotective effect in MA-induced neurotoxicity [52]. Furthermore, peroxynitrite has been shown to inhibit DAT and, therefore, inhibitory effects on DAT would favor cytosolic DA accumulation, which would lead to increased generation of ROS within dopaminergic neurons. It is also probable that early depletion of GSH might induce nitrosative damage to mitochondrial proteins, leading to activation of mitochondria-mediated cell death signaling events. Indeed, possible involvement of protein nitration of complex-1 inhibition by peroxynitrite in GSH depleted cells has been reported [53]. Also, NO might be the primary agent involved in mitochondrial dysfunction following acute GSH depletion in dopaminergic cells [54].

Another marker of increased oxidative stress is lipid peroxidation [9]. MA treatment resulted in increased levels of 4-HNE after 18 hours of MA treatment. Lipid peroxidation has been shown to persist for up to 24 hours after MA administration in rodents [55–57]. Also, GSH conjugates may combine with NO to form nitrosoglutathione and also with lipid peroxidation adducts 4-HNE [58, 59]. Alternatively, peroxynitrite is a potent oxidant species that has been found to cause lipid peroxidation independently [58, 60]. In the present study, treatment with NAC significantly reduced the levels of 4-HNE and apoptosis, indicating the importance of oxidative stress mechanisms in MA-induced cell death. In fact, in a recent study [57] MA was found to cause lipid peroxidation-mediated damage to Parkin and 26 S proteasome, thereby resulting in early loss of ubiquitin proteasomal (UPS) function [57]. Recently, we demonstrated that MA treatment impairs UPS function and triggers autophagy in both cell culture and animal models [61]. Furthermore, we demonstrated that genetic ablation or siRNA-mediated gene silencing of redox sensitive kinase, protein kinase c delta (PKC*δ*), conferred resistance against MA-induced dopaminergic apoptotic cell death in N27 cells, suggesting a causal role for PKC*δ* in MA-induced dopaminergic neurodegeneration. Additional studies from our laboratory also demonstrated that ROS is an integral component of the activation of a redox sensitive kinase PKC*δ* because superoxide scavenger MnTBAP attenuated Parkinsonian toxicant MPP-induced proteolytic activation of kinase and cell death [62] while prooxidants hydrogen peroxide [63] and 6-hydroxydopamine induced apoptosis through PKC*δ* activation in N27 dopaminergic cells [61]. Taken together, amelioration of MA-induced oxidative insult by NAC may be related to dampening of PKC*δ* proteolytic activation and associated apoptotic signaling events. Studies have demonstrated that acute administration of MA results in increased aldehyde accumulation in animal models of MA-induced neurodegeneration [9, 42, 64]. MA-induced oxidative stress is functionally linked to mitochondria-dependent apoptosis. Mitochondria serve as indispensable power houses of the cell and consume large amounts of oxygen in the mitochondrial respiratory chain pathway, resulting in production of a major source of ROS generation. Furthermore, a recent study [65] showed that autophagy is induced through oxidative inactivation of Atg4. Our results with MA-induced 3-NT and 4-HNE levels suggest that generation of nitrosylated oxidative species and lipid peroxides is presumably linked to activation of PKC delta-dependent mitochondria-mediated apoptotic cell death events, which may be central to MA-induced dopaminergic neurotoxicity. 

ROS-mediated events may not be the sole redox-related event involved in the regulation of autophagy. Other factors, such as GSH redox status, have also been shown to regulate autophagy [65, 66]. In the present study, we suggest that altered intracellular GSH content can modulate autophagy because (i) MA treatment was associated with an early depletion of GSH content; (ii) the addition of NAC replenished the intracellular level of GSH and partially prevented autophagy; and (iii) the depletion of cellular GSH by BSO increased the levels of autophagy. The fact that NAC pretreatment significantly increased GSH levels illustrates the significance of initial cellular redox state in influencing the cell response to MA exposure and supports the conclusion that observed changes may occur via a shift in intracellular redox state.

In conclusion, the present results reveal that loss of cellular levels of GSH is one of the pivotal mechanisms involved in MA-induced neurotoxicity and autophagy in mesencephalic dopaminergic neuronal cells and that treatment with NAC partially reverses MA-induced apoptotic cell death, possibly by replenishing GSH levels. Our results also indicate that MA-induced neurotoxicity is associated with increased 4-HNE levels and 3-NT adduct formation. Moreover, scavenging of free radicals such as RNS and ROS using NAC also partially attenuated MA-induced upregulation of autophagy. To the best of our knowledge, this is the first report demonstrating that NAC pretreatment can ameliorate MA-induced autophagy, highlighting the importance of redox status of the cell in MA-induced dopaminergic neurodegeneration. Further studies will be necessary to confirm the effect of NAC on redox status and autophagy, and the relevance to MA-induced dopaminergic degeneration in animal models.
